# Notes

**Published:** 1899-10

**Authors:** 


### NOTES.

Those who were in attendance at Nashville, and visited the famous Belle Mead Farm, will learn with much regret of the death of the famous running stallion "Iroquois." For many years "Iroquois" was the head of this stud, and had brought forth a number of sons whose fame as race-winners added lustre to the achievemenst of their noted sire. His death at twenty-one years of age, when he appeared to be just in the best of health and condition, added much to the loss suffered by General Jackson, his owner. For three weeks prior to his death he had been treated for rheumatism by one of Nashville's non-graduate veterinarians. His sudden death was followed by an autopsy by several of Nashville veterinarians and revealed the presence of an acute nephritis from which the animal had succumbed.

"Southern Hog Raising" is the subject-matter of a recent bulletin issued by the Department of Agriculture, which affords a deal of information as to the opportunities for the successful raising of swine in that territory. It will no doubt prove a successful field for the production of the bacon-hog, now so much desired for foreign shipment and home consumption.

